# Neighborhood-level sleep health and childhood opportunities

**DOI:** 10.3389/fpubh.2023.1307630

**Published:** 2024-02-05

**Authors:** Suzanne Gorovoy, Sydney Phan, Tommy K. Begay, Dora Valencia, Lauren Hale, Rebecca Robbins, William D. S. Killgore, Ariel A. Williamson, Michael Grandner

**Affiliations:** ^**1**^Sleep and Health Research Program, Department of Psychiatry, University of Arizona College of Medicine, Tucson, AZ, United States; ^2^Department of Family, Population and Preventative Medicine, Stony Brook University, Stony Brook, NY, United States; ^3^NYU Langone Medical Center, New York, NY, United States; ^4^SCAN Lab, Department of Psychiatry, University of Arizona College of Medicine, Tucson, AZ, United States; ^5^Children’s Hospital of Philadelphia, Philadelphia, PA, United States; ^6^University of Pennsylvania Perelman School of Medicine, Philadelphia, PA, United States

**Keywords:** sleep health disparities, child opportunity, neighborhood, short sleep, pediatric sleep

## Abstract

**Objectives:**

Regional sleep differences may reflect other important indicators of health and well-being. Examining sleep health at the regional level can help inform policies to improve population health. We examined the relationship between neighborhood-level adult sleep health (modeled in this study via adult sleep duration) and other health metrics and multiple indicators of child-relevant opportunity.

**Methods:**

Data were obtained from the “500 Cities” data collected by the CDC, including the proportion of the adult population in each tract that report obtaining at least 7 h of sleep. The Child Opportunity Index (COI) provides indices for “education,” “health and environment,” and “social and economic” domains, as well as a global score. When data were merged, 27,130 census tracts were included. Linear regression analyses examined COI associated with the proportion of the adult population obtaining 7 h of sleep.

**Results:**

Adult sleep duration was associated with global COI, such that for each additional percent of the population that obtains ≥ 7 h of sleep, COI increases by 3.6 points (95%CI[3.57, 3.64]). Each component of COI was separately related to adult sleep duration. All associations were attenuated but significant in adjusted analyses. In stepwise analyses, sleep health via adult sleep duration emerged as the strongest correlate of global COI, accounting for 57.2% of the variance (*p* < 0.0001). Similarly, when stepwise analyses examined each component of COI as dependent variable, sleep health consistently emerged as the most substantial correlate (all *p* < 0.0001).

**Conclusion:**

Community levels of sufficient sleep are associated with greater childhood opportunities, which itself is robustly associated with a wide range of health and economic outcomes. Future work can examine whether this association can develop into scalable interventions.

## Introduction

Insufficient sleep duration is associated with a range of negative health and social outcomes, including poorer educational performance in school and at work. It is associated with several leading causes of death in the United States (US), including cardiovascular disease, strokes, accidents, and diabetes (1) Current research suggests that there is a more direct relationship between insufficient sleep duration and negative health outcomes (1). Sleep is thus a more salient issue in the US and a major public health challenge that is associated with morbidity and mortality (2).

The multidimensional sleep health construct includes sleep both at an individual level and recognizes it within a larger socioecological context (3). To truly understand sleep health, it is critical to view it from a socioecological framework. In this framework, multiple environmental systems (e.g., individual, family, neighborhood, and community) interact to influence overall health, and specifically sleep health. Looking at physical, social, and environmental factors together may facilitate an understanding of the mechanisms underlying sleep disparities. One key component of sleep health is sleep duration. It may be an epidemiologically appropriate proxy for the larger sleep health construct, as recent epidemiologic studies have found that sleep duration is associated with obesity, diabetes, hypertension and mortality (4–6).

Neighborhood and related socioecological factors can affect sleep, and individuals of lower socioeconomic status (SES) and of racially and ethnically minoritized backgrounds that often reside within disadvantaged communities are more likely to experience poor sleep health as compared to their counterparts of higher SES and/or White backgrounds (3). Living within a lower-SES neighborhood has been associated with insomnia and other sleep disorders (7). These geographic areas are often characterized by environmental features that are deleterious to healthy sleep, including higher levels of ambient noise, bright lights, and air quality (8). Individuals living within socially adverse neighborhoods characterized by high levels of violence and low levels of social cohesion have both shorter sleep duration and poorer quality sleep, as compared with individuals residing within more cohesive and safe neighborhoods (9, 10).

The neighborhoods where children live, learn, and play strongly influence current health and subsequent outcomes, including economic mobility, educational attainment, and health. The Childhood Opportunity Index 2.0 (COI) measures neighborhood resources and conditions that are relevant for healthy child development (11). The creation of the COI is important because it permits the comparison of the neighborhood-level opportunities using a single metric. The COI 2.0 supports research exploring levels and inequalities in child opportunity across the country, or more specifically within a designated state, metropolitan area, city, or neighborhood. Indices such as the COI 2.0 may help hospitals and community health centers understand regional patterns of neighborhood-level determinants and health outcomes. Additionally, the index can be decomposed into specific indicators that may be particularly informative for understanding specific health outcomes, including sleep health. If changes in the socioecological environment can also impact sleep health, then examining neighborhood-level child health opportunities is a critical step in population-level efforts to improve child sleep health and, in turn, overall wellbeing.

Accordingly, the present study examined the relationship between neighborhood-level adult sleep health and other health metrics and multiple indicators of child-relevant opportunity. To examine these associations nationally, we used a composite index by neighborhood in each of the 500 largest US metropolitan areas. We hypothesized that there would be a positive relationship between increased adult sleep duration and children’s neighborhood opportunity. Analyses comparing the relevance of adult sleep duration on childhood neighborhood opportunity relative to other key health metrics were exploratory.

## Methods

Study data were merged from two existing datasets representing national-level data collected among adults in 2015: The Child Opportunity Index (COI) 2.0 dataset and the Centers for Disease Control and Prevention (CDC) PLACES dataset.

### Child Opportunity Index 2.0

The COI 2.0 dataset (11) was collected by the Institute for Child, Youth, and Family Policy at the Heller School for Social Policy and Management at Brandeis University. Data included 29 indicators aggregated from 2010 and (where available) 2015 data (11). These indicators are divided into three categories: “education,” “health and environment,” and “social and economic.” The education metric includes information on early childhood education centers, elementary school reading and math proficiency, secondary school graduation rates, advanced placement enrollment, college enrollment, school poverty, teacher experience, and adult education attainment. The health and environment metric includes information reflecting access to healthy food and green space, walkability, housing vacancy, hazardous waste sites, industrial pollutants, airborne microparticles, ozone concentration, extreme heat exposure, and health insurance coverage. The social & economic metric includes information on employment, commute duration, poverty rate, public assistance use, home ownership, high-skill employment, household income, and single-headed households. Each of these metrics is aggregated into a score ranging from 0 to 100, and then combined into a Global score (also ranging from 0 to 100). The COI has been used to inform research and guide policy (11).

#### COI2.0 data

The COI Global score was assessed as a raw score with a range of 0–100. Component scores (education, health and environment, and social and economic) were also analyzed as raw scores with a range of 0–100.

### CDC places

The CDC PLACES dataset is a result of an ongoing population surveillance collaboration between the CDC and the Robert Wood Johnson Foundation. It combines health metrics from the Behavioral Risk Factor Surveillance System (BRFSS) (12) with geocoded data in order to develop health surveillance estimates at the census tract level (10). The data used for the present analysis came from the 2017 dataset, which included the 500 largest cities in the USA (representing over 100 million US adults). This dataset was chosen, as it was the closest temporally to the COI dataset that included all relevant variables.

#### Sleep duration

The CDC PLACES data records, at the census tract level, the estimated percent of adult residents that report at least 7 h of habitual sleep duration. These data were collected using the item from the BRFSS, which has been used in numerous epidemiological studies of sleep health (CITE). This item asks respondents to record the amount of sleep they get in a typical 24-h period, recorded in whole numbers. Therefore, this metric reflects the estimated number of respondents who indicated 7 or more hours, relative to 6 or fewer hours. (Partial hours were not evaluated.)

#### Other health metrics

Other census tract-level health metrics obtained by the CDC PLACES dataset (based on BRFSS items) (12). Include: the percent of individuals in each census tract that have asthma, hypertension, hypercholesterolemia, kidney disease, obesity, arthritis, diabetes, chronic obstructive pulmonary disease (COPD), and coronary heart disease, number of teeth lost, the percent that have access to healthcare and that have had a medical and/or dental checkup in the past year, the percentage that are taking antihypertensive medications, the percentage that have engaged in colon health screening, cholesterol screening, pap tests, or mammography, the percentage that engaged in binge drinking or are current smokers, the percentage that reported poor overall mental and/or physical health in the past month, the percentage that engage in leisure time physical activity, the percent that have had a stroke and/or cancer, and the percentage of older adults ≥65 years that have engaged in a core set of health prevention activities for men and/or women (12).

### Data analyses

The COI 2.0 dataset includes more census tracts than the CDC PLACES dataset, which only included the 500 largest cities. Therefore, the data were merged by census tract, keeping only those in the COI 2.0 dataset that also had entries in the CDC PLACES dataset. Of note, the COI 2.0 dataset included 482 census tracts that are separately listed twice, in rare situations where the same census tract exists partially in two different neighborhoods, where that same census tract has two sets of COI values, reflecting the two separate neighborhoods. In these instances, the same CDC PLACES data was mapped onto both of those entries. Sensitivity analyses found that including or excluding those census tracts did not alter observed results.

To evaluate whether sleep duration is associated with COI global and component scores, linear regression analyses evaluated COI scores as dependent variable and the percent of census tracts that reported at least 7 h of sleep as the independent variable. A series of 5 models were run for each of 4 outcomes (COI global and 3 component scores, separately). The first model was unadjusted. The second model adjusted for population size within each census tract. The third model added the percent with access to healthcare and completion of a medical checkup in the past year. The fourth model included population size, the percent that reported healthcare access and a medical checkup, and the prevalence of obesity, hypertension, diabetes, and smoking. The final model included all of these variables as covariates, as well as the prevalence of those reporting poor mental health and poor physical health. Since many health metrics are related to each other, a series of forward stepwise regression analyses were computed to determine which health metrics accounted for unique variance in predicting COI global and component scores. Four separate models were run, with COI global and 3 component scores, separately as dependent variable. All models include census tract population size as covariate. Then, all health metrics, including sleep duration, were simultaneously added into the stepwise model. Using the forward stepwise approach, the variable that contributed the largest amount of unique variance was retained, and the model was re-run with the remaining variables to explain the remaining variance. This was conducted iteratively, until no additional variables contributed a statistically significant amount of unique variance (*p* > 0.05). All analyses were computed in STATA 17.0 (STATACORP, College Station, TX). All analyses are expressed as unstandardized regression coefficients and 95% Confidence Intervals (CI).

## Results

### Characteristics of the census tracts studied

The sample included *N* = 27,131 individual census tract-level observations. Characteristics of these census tracts are reported in Table 1. The average size of these census tracts was 3,786 individuals. Means and standard deviations for COI Global score, component scores, and percent of the population reporting a range of health-related outcomes is also reported in Table 1. Across census tracts, on average, the mean percentage of those reporting healthy sleep is about 63%.

**Table 1:** Characteristics of the census tracts in the sample.

Variable	%	SD
Census tracts	N	27,131
Population count	N	3,786 ± 1,945
Global COI score	Points (0 = 100)	43.3 ± 30.4
Health and environment COI component	Points (0 = 100)	40.9 ± 28.7
Social and economic COI component	Points (0 = 100)	43.7 ± 30.7
Education COI component	Points (0 = 100)	46.4 ± 30.3
Sleep health	% population	62.9 ± 6.4
Teeth lost	% population	16.3 ± 8.6
Access to healthcare	% population	17.5 ± 9.9
Asthma prevalence	% population	9.49 ± 1.74
Colon screening	% population	59.3 ± 9.4
Dental checkup	% population	59.8 ± 13.2
Leisure time physical activity	% population	27.0 ± 8.5
Core prevention for women	% population	28.6 ± 7.3
Cholesterol screening	% population	72.6 ± 8.4
Arthritis prevalence	% population	21.8 ± 6.2
Pap testing	% population	78.9 ± 6.4
Annual medical checkup	% population	68.4 ± 6.2
Coronary heart disease prevalence	% population	5.64 ± 2.12
Stroke prevalence	% population	3.17 ± 1.53
Smoking prevalence	% population	18.4 ± 6.5
Binge drinking prevalence	% population	16.8 ± 3.9
Physical health	% population	12.9 ± 4.4
Cancer prevalence	% population	5.58 ± 1.81
Core prevention for men	% population	29.9 ± 6.5
Kidney disease prevalence	% population	2.74 ± 0.82
Mental health	% population	12.9 ± 3.5
Diabetes prevalence	% population	10.7 ± 4.3
Antihypertensive use	% population	72.2 ± 7.7
Hypertension prevalence	% population	30.5 ± 8.3
Obesity prevalence	% population	29.7 ± 8.0

### Association between neighborhood sleep duration and COI global and component scores

Results of regression analyses examining relationships between COI Global and component scores are reported in Table 2. In unadjusted analyses, each percent increase in population within the census tract that reports adequate adult sleep duration is associated with an increase in the COI Global score of approximately 3.6 points; it was also associated with an increase of about 3.1, 2.2, and 3.6 points in the Education, Health and Environment, and Social and Economic components, respectively. Additional models reported in Table 2 included adjustment for population size within each census tract, access to healthcare, health status, and other health-related behaviors. In the fully adjusted model, the percent that obtained adequate sleep duration was still associated with the Global score as well as all three component scores, though the effects were attenuated. Across all models, for all COI variables, all results were statistically significant (*p* < 0.0001).

**Table 2:** Association between childhood opportunity index global score and components with sleep health (all *P* < 0.0001).

	*B*	95% CI
Unadjusted		
Global	3.603	(3.567, 3.640)
Education	3.055	(3.012, 3.098)
Health and environment	2.234	(2.188, 2.280)
Social and economic	3.606	(3.568, 3.643)
Population-adjusted*		
Global	3.600	(3.563, 3.636)
Education	3.043	(3.000, 3.086)
Health and environment	2.209	(2.163, 2.255)
Social and economic	3.610	(3.572, 3.647)
Population + access adjusted**		
Global	2.628	(2.587, 2.669)
Education	2.294	(2.240, 2.348)
Health and environment	1.003	(0.948, 1.058)
Social and economic	2.794	(2.750, 2.838)
Population + health adjusted***		
Global	1.582	(1.533, 1.632)
Education	1.259	(1.193, 1.326)
Health and environment	0.587	(0.516, 0.658)
Social and economic	1.768	(1.714, 1.823)
Fully adjusted****		
Global	1.177	(1.130, 1.223)
Education	0.858	(0.797, 0.919)
Health and environment	0.190	(0.124, 0.257)
Social and economic	1.388	(1.337, 1.439)

*Adjusted for population within the census tract. **Adjusted for population within the census tract and % access to healthcare and % annual checkup. ***Adjusted for population within the census tract and % access to healthcare, % annual checkup, % hypertension, % diabetes, % obesity, and % smoking. ****Adjusted for population within the census tract and % access to healthcare, % annual checkup, % hypertension, % diabetes, % obesity, % smoking, and % with poor mental and physical health.

### Stepwise analysis

To investigate the relative importance of adult sleep duration relative to other health metrics, a stepwise analysis was computed, which included population of each census tract as a covariate, and all possible health metrics as independent variables. In examining Global COI, sleep emerged as the variable that explained the most unique variance. Results of this analysis are reported in Table 3. Adult sleep duration alone accounted for 57.18% of the variance of Global COI. The other variables that contributed more than 1% of total variance as they were added sequentially included (in order): teeth lost, access to healthcare, asthma prevalence, colon screening, and dental checkup. All variables contributed unique variance (*p* < 0.05) except COPD prevalence and mammography (see Table 3).

**Table 3:** Stepwise analysis examining variables associated with child opportunity index global score.

	% variance explained	Coefficient	95% CI	*p* (final model)
Population count	0.05%	0.00001	(−0.00006, 0.00009)	0.744
Sleep health	57.18%	0.4672	(0.399, 0.536)	<0.0005
Teeth lost	15.53%	1.29242	(1.217, 1.368)	<0.0005
Access to healthcare	2.98%	0.31501	(0.260, 0.370)	<0.0005
Asthma prevalence	1.42%	−2.1992	(−2.489, −1.910)	<0.0005
Colon screening	1.34%	−0.04466	(−0.115, 0.026)	0.214
Dental checkup	1.87%	1.63307	(1.569, 1.698)	<0.0005
Leisure time physical activity	0.55%	−0.37218	(−0.446, −0.298)	<0.0005
Core prevention for women	0.73%	0.70574	(0.646, 0.765)	<0.0005
Cholesterol screening	0.53%	1.4068	(1.337, 1.477)	<0.0005
Arthritis prevalence	0.53%	−0.66472	(−0.792, −0.538)	<0.0005
Pap testing	0.79%	−1.24246	(−1.306, −1.179)	<0.0005
Annual medical checkup	0.26%	−0.40222	(−0.466, −0.338)	<0.0005
Coronary heart disease	0.09%	−7.07621	(−7.565, −6.587)	<0.0005
Stroke prevalence	0.30%	5.95075	(5.320, 6.582)	<0.0005
Smoking prevalence	0.07%	−0.51081	(−0.602, −0.419)	<0.0005
Binge drinking prevalence	0.03%	0.37454	(0.302, 0.448)	<0.0005
Physical health	0.05%	2.19815	(1.903, 2.493)	<0.0005
Cancer prevalence	0.06%	2.71587	(2.345, 3.086)	<0.0005
Core prevention for men	0.12%	0.39401	(0.331, 0.457)	<0.0005
Kidney disease prevalence	0.03%	−5.97364	(−7.053, −4.895)	<0.0005
Mental health	0.05%	−1.02825	(−1.280, −0.776)	<0.0005
Diabetes prevalence	0.02%	1.11811	(0.858, 1.378)	<0.0005
Antihypertensive use	0.02%	−0.1415	(−0.199, −0.084)	<0.0005
Hypertension prevalence	<0.005%	−0.22221	(−0.327, −0.117)	<0.0005
Obesity prevalence	0.01%	0.0856	(0.024, 0.147)	0.007
COPD prevalence	N/A	N/A	N/A	N/A
Mammography	N/A	N/A	N/A	N/A

In supplementary analyses, separate stepwise regression analyses were computed for each of the component scores of the COI. Results are reported in Supplementary Table S1 (Education component), Supplementary Table S2 (Health and Environment component), and Supplementary Table S3 (Social and Economic component). In all three cases, sleep duration emerged as the variable that explained the most unique variance of each of the three components, uniquely accounting for 41.8, 25.0, and 56.7% of the variance in the Education, Health and Environment, and Social and Economic components, respectively. Of note, in the final model for Health and Environment, although sleep duration added the most unique variance and was included as the first variable in the model, it was nonsignificant, with reduced unique variance explained in the final model, after the inclusion of all other variables.

## Discussion

This study evaluated the relationship between adult neighborhood-level sleep duration and other key adult health metrics on multiple indicators of child development opportunities via the Childhood Opportunity Index (COI) global and domain scores. We found that among a variety of health indicators, sleep duration was the strongest individual variable correlated with overall neighborhood-level child development opportunities, accounting for 57.2% of the variance in the COI global score. In addition, neighborhood-level sleep duration was the most significant predictor of each individual component of the COI, which is made up of “education,” “health and environment,” and “social and economic” domain scores.

Neighborhood-level sleep health, modeled in this study via sleep duration, was the most robust health correlate of the factors that are associated with neighborhood opportunities for positive child growth and success. Across a variety of health indicators, neighborhood-level sleep duration was the strongest predictor of opportunity for children in a neighborhood, in terms of the Childhood Opportunity Index global score. Additionally, neighborhood-level sleep duration was the strongest correlate of each of the three domain scores that comprise the COI global score. These findings suggest that neighborhood-level sleep duration may reflect neighborhood-level resources, including its positive educational opportunities, environmental health and safety, and social supports.

These findings contribute to a growing body of research underscoring sleep as a crucial health indicator on that could be leveraged to reduce broader health inequities (13). Addressing sleep disparities at the neighborhood level may help to reduce other health disparities and improve well-being. Neighborhood-level sleep health promotion efforts could include changes to local policies, such as middle and high school start times, as well as improvements to features of the neighborhood social environment (e.g., social cohesion) and physical characteristics (e.g., ambient light pollution; green space).

Neighborhood level features of the environment surrounding the home have a substantial impact on sleep health. The COI includes other child opportunity indicators within its three domains that represent neighborhood-level features that have also been previously shown in the literature to be associated with adult sleep health outcomes. One COI indicator under the Health and Environmental Health Opportunity domain that has an established association with sleep health would be proximity to parks and open spaces. Observational studies have shown that adults living in neighborhoods with access to green space or natural water features have a lower likelihood of insufficient sleep (14, 15). Tree canopy was associated with longer weekday sleep duration (16). Green spaces may promote walking and other healthy behaviors, lower stress levels, and improve mental health which could facilitate healthy sleep. In the Social and Economic Opportunity domain of the COI, another indicator that is associated with adult sleep health is socioeconomic status. DeSantis et al. found that neighborhood socioeconomic status was statistically significantly associated with self-reported daytime sleepiness such that, for every one-standard deviation decrease in SES, participants were 6% sleepier (95%CI: 0.11, 0.01) (17). Adult educational attainment is an indicator of COI under the Educational Opportunity domain shown to have an association with adult sleep health. A study by Grandner et al., found that individuals with lower levels of educational attainment were more likely to report long sleep latency and snoring (18). In a study comparing compared the likelihood of insomnia and insomnia-related health consequences among individuals of different socioeconomic status, results indicated that individuals of lower individual and household education were significantly more likely to experience insomnia even after researchers accounted for ethnicity, gender, and age. Additionally, individuals with fewer years of education, particularly those who had dropped out of high school, experienced greater subjective impairment because of their insomnia (19).

Study findings also highlight the importance of addressing sleep at a family level, given that this study focused on adults’ sleep duration and that prior research demonstrates an overwhelming majority of school-aged children and adolescents obtain insufficient sleep. Sleep health behaviors (such as sleep duration) are emerging as family-level health behaviors. Family members influence one another’s sleep through their physical presence and through psychological and emotional mechanisms. Family members’ sleep patterns may also be coregulated (20). The negative relationship between inadequate sleep duration and a host of poorer child outcomes, including physical health, mood/well-being, academic, and neurocognitive outcomes, is well documented in the pediatric sleep literature (21–23). More research is needed to examine the effect of adult sleep behaviors (such as sleep duration) on sleep behaviors (such as sleep duration) in children.

Future sleep health and public health research will benefit from recognizing the limitations of individual-level sleep health interventions in isolation and shift toward developing sleep health interventions that address multiple socioecological levels, including neighborhood characteristics and related health policies. Future intervention research in sleep health promotion would benefit from focusing on developing and disseminating place-conscious, evidence-based, effective, and scalable interventions that account for the role of multiple socioecological systems that interact to influence individual child development. Interventions that improve neighborhood-level characteristics (e.g., safety, air quality) should also be examined, as they may further contribute to improved sleep health within a community. Broader scale public health interventions (e.g., sleep health education awareness campaigns) implemented in coordination with relevant advisory groups, including families living in the neighborhoods where interventions are implemented, have the potential to improve sleep health at the population level, and in tandem improve childhood outcomes.

### Limitations

The study was limited to data from two existing databases representing national-level data around 2015. While the COI 2.0 is a comprehensive index with three domains, data are only available for two time points, one in 2010 and one in 2015. While the two time points may be comparable, these data cannot currently be used in longitudinal analyses examining how social determinants of health such as neighborhood-level sufficient sleep may change over period of time. However, understanding the long-term contribution of neighborhood contexts to child health offers a deeper opportunity to influence population health and well-being through targeted neighborhood-level interventions, resource allocation, understanding the causes of neighborhood inequity, and informing place-based interventions.

It is important to note that other unmeasured factors may contribute to neighborhood-level sleep health. For instance, exposure to adverse childhood experiences (ACEs) have been associated with sleep health. ACEs have also been associated with increased odds of short sleep duration in a dose–response manner, with some of these linkages extending up to 50 years beyond initial ACEs exposure (24).

The COI uses census-tract level data, which may not align with how individuals in a neighborhood perceive their neighborhood boundaries. This factor is important to recognize, as this may impact how neighborhood-level sleep health interventions are designed to address health inequity are designed and implemented.

There were also limitations in how sleep health was ascertained. First, these data include only adult self-reported estimates of habitual sleep duration, without corroborating prospective, e.g., sleep diary and/or objective (e.g., actigraphy) estimates and may therefore be subject to error. Second, sleep health was measured by habitual sleep duration, which represents only one domain of sleep health (3). No other census tract-level estimates of sleep health exist for other metrics, however. Of note, habitual sleep duration is the metric that is most consistently associated with health outcomes at the individual level (13) and is the metric used in the American Heart Association’s “Life’s Essential 8” criteria for heart health” (25). Future population surveillance measures should include other aspects of sleep health in addition to sleep duration.

## Conclusion and future directions

The present study examined relationships between neighborhood-level sleep health (modeled in this study via adult sleep duration) and neighborhood-level child development opportunities. Overall, sleep health (represented as the proportion of the adult population that reported the recommended 7 or more hours of habitual nightly sleep duration) was associated with COI global and component scores, even after adjustment for other regional characteristics. Further, adult sleep health was the strongest unique correlate of the global score as well as all three component scores. Findings suggests that a neighborhood’s sleep health (modeled in this study via adult sleep duration) is an important neighborhood health indicator that is related not only to the health of the adults in that neighborhood but also the conditions needed to support its children. Future work should extend these findings by exploring other domains of sleep health, examining sleep health as a source of neighborhood resilience, and evaluating community-engaged interventions to promote sleep health at the neighborhood level.

## Data availability statement

The original contributions presented in the study are included in the article/Supplementary material, further inquiries can be directed to the corresponding author.

## Author contributions

SG: Conceptualization, Methodology, Writing – original draft, Writing – review & editing. SP: Conceptualization, Data curation, Writing – review & editing. TB: Writing – review & editing. DV: Writing – review & editing. LH: Writing – review & editing. RR: Writing – review & editing. WK: Writing – review & editing. AW: Writing – review & editing. MG: Conceptualization, Formal analysis, Methodology, Writing – original draft, Writing – review & editing.
