# Supplementary material for: Neighborhood-level sleep health and childhood opportunities
Source: Front Public Health. 2024 Feb 5;11:1307630. doi: 10.3389/fpubh.2023.1307630 (PMC10875035; doi:10.3389/fpubh.2023.1307630)
Supplement: Supplementary file 1 [file Data_Sheet_1.docx]

**Supplementary Table 1. Stepwise Analysis Examining Variables Associated with Child Opportunity Index Education Component Score**

|  | **Coefficient** | **95% CI** | **p (final model)** |
| --- | --- | --- | --- |
| Population Count | 0.0001568 | (0.00004, 0.00027) | 0.006 |
| Sleep Health | 1.265543 | (1.170, 1.361) | <0.0001 |
| Teeth Lost | 0.7971905 | (0.684, 0.910) | <0.0001 |
| Dental Checkup | 1.055041 | (0.965, 1.145) | <0.0001 |
| Obesity Prevalence | -0.4167683 | (-0.508, -0.326) | <0.0001 |
| Asthma Prevalence | -2.633571 | (-3.039, -2.228) | <0.0001 |
| Arthritis Prevalence | -2.24323 | (-2.420, -2.067) | <0.0001 |
| Hypertension Prevalence | 0.4824074 | (0.330, 0.635) | <0.0001 |
| Pap Testing | -2.160374 | (-2.266, -2.055) | <0.0001 |
| Cholesterol Screening | 2.046671 | (1.941, 2.153) | <0.0001 |
| Core Prevention for Men | 0.9010239 | (0.821, 0.981) | <0.0001 |
| Stroke Prevalence | 7.244651 | (6.253, 8.236) | <0.0001 |
| Smoking Prevalence | -1.076987 | (-1.221, -0.933) | <0.0001 |
| Mental Health | 2.204797 | (1.816, 2.594) | <0.0001 |
| Coronary Heart Disease | -8.347348 | (-9.130, -7.565) | <0.0001 |
| COPD Prevalence | 2.940363 | (2.407, 3.473) | <0.0001 |
| Leisure Time Physical Activity | 0.7344858 | (0.629, 0.840) | <0.0001 |
| Mammography | 0.2655491 | (0.167, 0.364) | <0.0001 |
| Kidney Disease Prevalence | 10.60541 | (9.082, 12.129) | <0.0001 |
| Antihypertensive Use | -0.4626066 | (-0.554, -0.371) | <0.0001 |
| Physical Health | -1.511642 | (-1.953, -1.071) | <0.0001 |
| High Cholesterol | 0.5141444 | (0.365, 0.663) | <0.0001 |
| Colon Screening | 0.3856101 | (0.279, 0.492) | <0.0001 |
| Binge Drinking Prevalence | 0.2498883 | (0.143, 0.356) | <0.0001 |
| Diabetes Prevalence | -0.5953954 | (-0.937, -0.253) | 0.001 |
| Annual Medical Checkup | -0.1596379 | (-0.265, -0.054) | 0.003 |
| Access to Healthcare | N/A | N/A | N/A |
| Cancer Prevalence | N/A | N/A | N/A |
| Core Prevention for Women | N/A | N/A | N/A |

**Supplementary Table 2. Stepwise Analysis Examining Variables Associated with Child Opportunity Index Health and Environment Component Score**

|  | **Coefficient** | **95% CI** | **p (final model)** |
| --- | --- | --- | --- |
| Population Count | 0.0008041 | (0.001, 0.001) | <0.0001 |
| Sleep Health | 0.0102439 | (-0.082, 0.102) | 0.827 |
| Teeth Lost | 0.7715022 | (0.660, 0.883) | <0.0001 |
| Access to Healthcare | -0.3379259 | (-0.415, -0.261) | <0.0001 |
| Dental Checkup | 1.842803 | (1.742, 1.944) | <0.0001 |
| Obesity Prevalence | -1.155434 | (-1.250, -1.061) | <0.0001 |
| Physical Health | 9.147102 | (8.711, 9.583) | <0.0001 |
| COPD Prevalence | -7.862784 | (-8.419, -7.307) | <0.0001 |
| Mammography | -0.9912259 | (-1.093, -0.890) | <0.0001 |
| Colon Screening | 0.9402375 | (0.825, 1.056) | <0.0001 |
| Smoking Prevalence | 0.6156718 | (0.461, 0.770) | <0.0001 |
| Pap Testing | -0.9586336 | (-1.050, -0.867) | <0.0001 |
| Mental Health | -2.961293 | (-3.348, -2.575) | <0.0001 |
| Coronary Heart Disease | -7.943048 | (-8.729, -7.157) | <0.0001 |
| Arthritis Prevalence | 1.135806 | (0.938, 1.334) | <0.0001 |
| Binge Drinking Prevalence | 0.7558588 | (0.639, 0.873) | <0.0001 |
| Diabetes Prevalence | 2.155011 | (1.766, 2.544) | <0.0001 |
| Kidney Disease Prevalence | -10.47143 | (-12.122, -8.821) | <0.0001 |
| Cancer Prevalence | 2.966963 | (2.394, 3.540) | <0.0001 |
| Asthma Prevalence | 1.644398 | (1.208, 2.081) | <0.0001 |
| Core Prevention for Women | 0.4827668 | (0.385, 0.580) | <0.0001 |
| Stroke Prevalence | 3.503993 | (2.536, 4.472) | <0.0001 |
| Hypertension Prevalence | -0.4601567 | (-0.625, -0.295) | <0.0001 |
| Core Prevention for Men | -0.2549166 | (-0.357, -0.153) | <0.0001 |
| Annual Medical Checkup | N/A | N/A | N/A |
| Antihypertensive Use | N/A | N/A | N/A |
| Cholesterol Screening | N/A | N/A | N/A |
| High Cholesterol | N/A | N/A | N/A |

**Supplementary Table 3. Stepwise Analysis Examining Variables Associated with Child Opportunity Index Social and Economic Component Score**

|  | **Coefficient** | **95% CI** | **p (final model)** |
| --- | --- | --- | --- |
| Population Count | -0.0002362 | (-0.00032, -0.00015) | <0.0001 |
| Sleep Health | 0.4880408 | (0.410, 0.566) | <0.0001 |
| Teeth Lost | 1.337258 | (1.251, 1.424) | <0.0001 |
| Access to Healthcare | 0.4381188 | (0.375, 0.501) | <0.0001 |
| Asthma Prevalence | -2.123911 | (-2.455, -1.792) | <0.0001 |
| Colon Screening | -0.3225575 | (-0.403, -0.242) | <0.0001 |
| Core Prevention for Women | 0.9127055 | (0.844, 0.982) | <0.0001 |
| Dental Checkup | 1.445472 | (1.372, 1.519) | <0.0001 |
| Leisure Time Physical Activity | -0.5721058 | (-0.657, -0.488) | <0.0001 |
| Cholesterol Screening | 1.323895 | (1.241, 1.406) | <0.0001 |
| Coronary Heart Disease | -5.549298 | (-6.189, -4.910) | <0.0001 |
| Pap Testing | -0.8188749 | (-0.896, -0.742) | <0.0001 |
| Annual Medical Checkup | -0.4116305 | (-0.485, -0.339) | <0.0001 |
| Stroke Prevalence | 4.64273 | (3.896, 5.390) | <0.0001 |
| Mental Health | -1.088896 | (-1.381, -0.797) | <0.0001 |
| Kidney Disease Prevalence | -8.842335 | (-10.091, -7.593) | <0.0001 |
| Smoking Prevalence | -0.6694397 | (-0.783, -0.556) | <0.0001 |
| Obesity Prevalence | 0.452948 | (0.381, 0.524) | <0.0001 |
| Binge Drinking Prevalence | 0.4061587 | (0.322, 0.490) | <0.0001 |
| Physical Health | 1.325281 | (0.985, 1.665) | <0.0001 |
| Cancer Prevalence | 2.719002 | (2.297, 3.141) | <0.0001 |
| Arthritis Prevalence | -0.4159748 | (-0.561, -0.271) | <0.0001 |
| Core Prevention for Men | 0.2861415 | (0.215, 0.358) | <0.0001 |
| Diabetes Prevalence | 1.35906 | (1.057, 1.661) | <0.0001 |
| Hypertension Prevalence | -0.32895 | (-0.450, -0.208) | <0.0001 |
| COPD Prevalence | 0.5769658 | (0.164, 0.990) | 0.006 |
| Antihypertensive Use | -0.080562 | (-0.146, -0.015) | 0.016 |
| Mammography | N/A | N/A | N/A |
| High Cholesterol | N/A | N/A | N/A |
